# (*E*)-6-Methyl-3-(2-methyl­benzyl­idene)­chroman-2-one

**DOI:** 10.1107/S1600536812005624

**Published:** 2012-02-24

**Authors:** S. Vijayakumar, S. Murugavel, D. Kannan, M. Bakthadoss

**Affiliations:** a Department of Physics, Sri Balaji Chokkalingam Engineering College, Arni, Thiruvannamalai 632 317, India; b Department of Physics, Thanthai Periyar Government Institute of Technology, Vellore 632 002, India; c Department of Organic Chemistry, University of Madras, Maraimalai Campus, Chennai 600 025, India

## Abstract

In the title compound, C_18_H_16_O_2_, the heterocyclic ring of the chroman-2-one system adopts a slightly distorted screw-boat conformation. The dihedral angle between the least-squares planes of the coumarin ring system and the benzene ring is 67.5 (1)°. The crystal packing features C—H⋯O hydrogen bonds, which link the mol­ecules into centrosymmetric *R_2_^2^*(8) dimers, and C—H⋯π inter­actions.

## Related literature

For the biological activity of coumarins, see: Sharma *et al.* (2005 ▶); Iqbal *et al.* (2009 ▶); Siddiqui *et al.* (2009 ▶); Vyas *et al.* (2009 ▶); Rollinger *et al.* (2004 ▶); Brühlmann *et al.* (2001 ▶). For ring-puckering parameters, see: Cremer & Pople (1975 ▶). For closely related structures, see: Choi & Kim (2010 ▶); Peng *et al.* (2012 ▶).
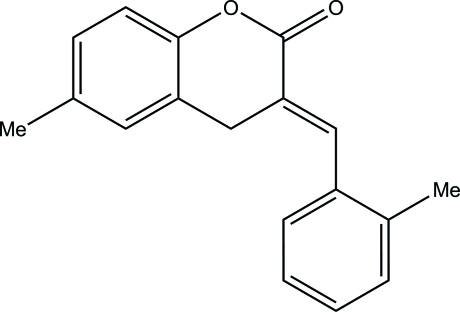


## Experimental

### 

#### Crystal data

C_18_H_16_O_2_
*M* *_r_* = 264.31
Monoclinic, 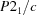

*a* = 9.1331 (2) Å
*b* = 17.8838 (5) Å
*c* = 9.6443 (3) Å
β = 118.056 (1)°
*V* = 1390.14 (7) Å^3^
*Z* = 4
Mo *K*α radiation
μ = 0.08 mm^−1^
*T* = 293 K
0.21 × 0.18 × 0.16 mm

#### Data collection

Bruker APEXII CCD diffractometer
Absorption correction: multi-scan (*SADABS*; Sheldrick, 1996 ▶) *T*_min_ = 0.983, *T*_max_ = 0.987
17986 measured reflections
4246 independent reflections
2882 reflections with *I* > 2σ(*I*)
*R*_int_ = 0.026

#### Refinement

*R*[*F* ^2^ > 2σ(*F* ^2^)] = 0.047
*wR*(*F* ^2^) = 0.152
*S* = 1.00
4246 reflections
183 parameters
H-atom parameters constrained
Δρ_max_ = 0.27 e Å^−3^
Δρ_min_ = −0.17 e Å^−3^

### 

Data collection: *APEX2* (Bruker, 2004 ▶); cell refinement: *APEX2* and *SAINT* (Bruker, 2004 ▶); data reduction: *SAINT* and *XPREP* (Bruker, 2004 ▶); program(s) used to solve structure: *SHELXS97* (Sheldrick, 2008 ▶); program(s) used to refine structure: *SHELXL97* (Sheldrick, 2008 ▶); molecular graphics: *ORTEP-3* (Farrugia, 1997 ▶); software used to prepare material for publication: *SHELXL97* and *PLATON* (Spek, 2009 ▶).

## Supplementary Material

Crystal structure: contains datablock(s) global, I. DOI: 10.1107/S1600536812005624/gk2454sup1.cif

Structure factors: contains datablock(s) I. DOI: 10.1107/S1600536812005624/gk2454Isup2.hkl

Supplementary material file. DOI: 10.1107/S1600536812005624/gk2454Isup3.cml

Additional supplementary materials:  crystallographic information; 3D view; checkCIF report

## Figures and Tables

**Table 1:** Hydrogen-bond geometry (Å, °) *Cg* is the centroid of the C1–C6 ring.

*D*—H⋯*A*	*D*—H	H⋯*A*	*D*⋯*A*	*D*—H⋯*A*
C11—H11⋯O1^i^	0.93	2.53	3.437 (2)	167
C14—H14⋯*Cg*^ii^	0.93	2.88	3.611 (2)	137
C18—H18*A*⋯*Cg*^iii^	0.96	2.74	3.490 (2)	136

Symmetry codes: (i) 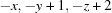; (ii) 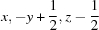; (iii) 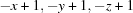.

**Fig. 1.**
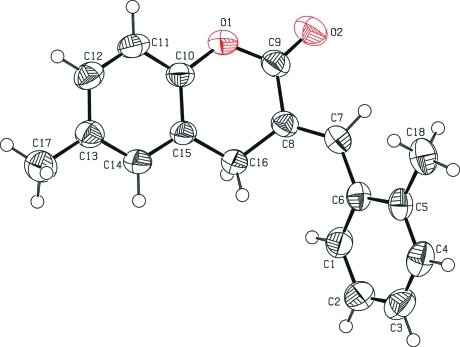
The molecular structure of the title compound with the atom numbering scheme. Displacement ellipsoids are drawn at the 50% probability level. H atoms are presented as a small spheres of arbitrary radius.

**Fig. 2.**
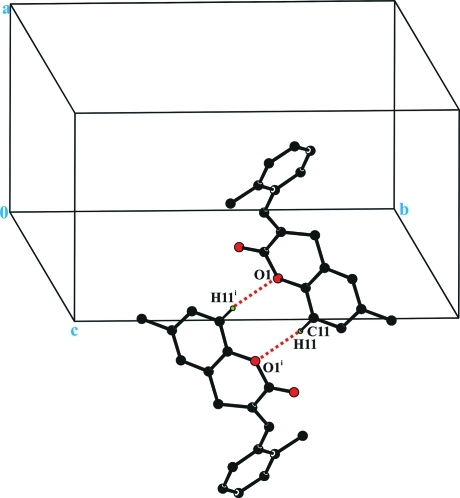
Part of the crystal structure of the title compound showing C—H···O intermolecular hydrogen bonds (dotted lines) generating R22(8) centrosymmetric dimer. [Symmetry code: (i) -x, 1-y, 2-z].

**Fig. 3.**
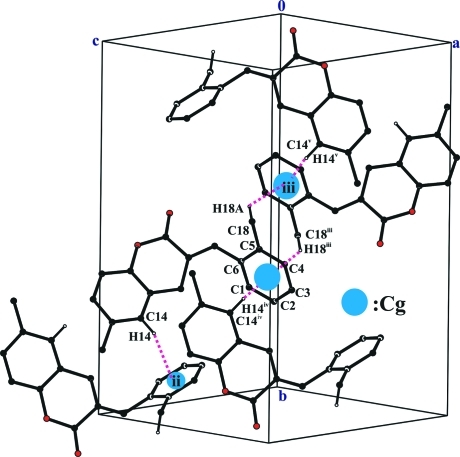
A view of the C-H···π interactions(dotted lines) in the crystal stucture of the title compound. H atoms not participating in hydrogen-bonding were omitted for clarity. [Symmetry codes: (ii) x, 3/2-y, 1/2+z; (iii) 1-x, 1-y, 1-z; (iv) x, 3/2-y, -1/2+z; (v) 1-x, -1/2-y, 3/2-z].

## supplementary crystallographic information

### Comment

Coumarins are very well known for their biological activity, such as antioxidant (Sharma *et al.*, 2005), antiamoebic (Iqbal *et al.*, 2009), anticonvulsant (Siddiqui *et al.*, 2009), antimicrobial (Vyas *et al.*, 2009) and inhibitions of acetylcholinesterase and monoamine oxidase (Rollinger *et al.*, 2004; Brühlmann *et al.*, 2001). In view of this importance, the crystal structure of the title compound has been carried out and the results are presented here.

Fig. 1. shows a displacement ellipsoid plot of of the title compound, with the atom numbering scheme. The pyranone ring adopts a distorted screw-boat conformation as indicated from the puckering parameters (Cremer & Pople, 1975): Q = 0.3229 (15) Å, θ = 72.1 (3)° and φ = 154.8 (3)°. The dihedral angle between the least-squares planes of the coumarine ring system (O1/C8–C16) and the benzene ring (C1–C6) is 67.5 (1)°. The geometric parameters of the title molecule agree well with those reported for similar structures (Choi & Kim, 2010; Peng *et al.*, 2012).

The crystal packing (Fig. 2) is stabilized by intermolecular C—H···O hydrogen bonds. The molecules at *x, y, z* and *-x, 1-y, 2-z* are linked by C11—H11···O1 hydrogen bonds through cyclic centrosymmetric *R_2_^2^*(8) motifs (See Table 1; first entry). The crystal packing (Fig. 3) is further stabilized by C—H···π interactions (See Table 1; second and third entry, Cg is the centroid of the C1–C6 benzene ring).

### Experimental

A solution of methyl 2-[hydroxy(2-methylphenyl)methyl]prop-2-enoate (0.206 g, 1 mmol) and *p*-cresol (0.108 g, 1 mmol) in CH_2_Cl_2_ solvent was allowed to cool at 0^o^C. To this solution, concentrated H_2_SO_4_ (0.98 g, 1 mmol) was added, and then stirred well at room temperature. After the completion of the reaction, as indicated by TLC, the reaction mixture was quenched with 1 M sodium bicarbonate and then extracted with CH_2_Cl_2_. The combined organic layers were washed with brine (2 X 10 ml) and dried over anhydrous sodium sulfate. The organic layer was evaporated and the residue was purified by column chromatography on silicagel (100–200) mesh, using ethylacetate and hexanes (1:9) as solvents. The pure title compound was obtained as a colourless solid (0.169 g, 64.5% yield, m.p. 407–409K). Recrystallization was carried out using ethyl acetate as solvent.

### Refinement

All the H atoms were positioned geometrically, with C–H = 0.93–0.97 Å and constrained to ride on their parent atom, with *U*_iso_(H) =1.5*U*_eq_ for methyl H atoms and 1.2*U*_eq_(C) for other H atoms.

### Crystal data

**Table:**

C_18_H_16_O_2_	*F*(000) = 560
*M**_r_* = 264.31	*D*_x_ = 1.263 Mg m^−^^3^
Monoclinic, *P*2_1_/*c*	Mo *K*α radiation, λ = 0.71073 Å
Hall symbol: -P 2ybc	Cell parameters from 4254 reflections
*a* = 9.1331 (2) Å	θ = 2.3–30.6°
*b* = 17.8838 (5) Å	µ = 0.08 mm^−^^1^
*c* = 9.6443 (3) Å	*T* = 293 K
β = 118.056 (1)°	Block, colourless
*V* = 1390.14 (7) Å^3^	0.21 × 0.18 × 0.16 mm
*Z* = 4	

### Data collection

**Table:**

Bruker APEXII CCD diffractometer	4246 independent reflections
Radiation source: fine-focus sealed tube	2882 reflections with *I* > 2σ(*I*)
Graphite monochromator	*R*_int_ = 0.026
Detector resolution: 10.0 pixels mm^-1^	θ_max_ = 30.6°, θ_min_ = 2.3°
ω scans	*h* = −13→13
Absorption correction: multi-scan (*SADABS*; Sheldrick, 1996)	*k* = −25→24
*T*_min_ = 0.983, *T*_max_ = 0.987	*l* = −13→13
17986 measured reflections	

### Refinement

**Table:**

Refinement on *F*^2^	Primary atom site location: structure-invariant direct methods
Least-squares matrix: full	Secondary atom site location: difference Fourier map
*R*[*F*^2^ > 2σ(*F*^2^)] = 0.047	Hydrogen site location: inferred from neighbouring sites
*wR*(*F*^2^) = 0.152	H-atom parameters constrained
*S* = 1.00	*w* = 1/[σ^2^(*F*_o_^2^) + (0.0777*P*)^2^ + 0.1937*P*] where *P* = (*F*_o_^2^ + 2*F*_c_^2^)/3
4246 reflections	(Δ/σ)_max_ < 0.001
183 parameters	Δρ_max_ = 0.27 e Å^−^^3^
0 restraints	Δρ_min_ = −0.17 e Å^−^^3^

### Special details

**Table:**

Geometry. All esds (except the esd in the dihedral angle between two l.s. planes) are estimated using the full covariance matrix. The cell esds are taken into account individually in the estimation of esds in distances, angles and torsion angles; correlations between esds in cell parameters are only used when they are defined by crystal symmetry. An approximate (isotropic) treatment of cell esds is used for estimating esds involving l.s. planes.
Refinement. Refinement of F^2^ against ALL reflections. The weighted R-factor wR and goodness of fit S are based on F^2^, conventional R-factors R are based on F, with F set to zero for negative F^2^. The threshold expression of F^2^ > 2sigma(F^2^) is used only for calculating R-factors(gt) etc. and is not relevant to the choice of reflections for refinement. R-factors based on F^2^ are statistically about twice as large as those based on F, and R- factors based on ALL data will be even larger.

### Fractional atomic coordinates and isotropic or equivalent isotropic displacement parameters (Å^2^)

**Table:**

	*x*	*y*	*z*	*U*_iso_*/*U*_eq_	
C1	0.53567 (17)	0.64217 (8)	0.67561 (18)	0.0510 (3)	
H1	0.5781	0.6520	0.7823	0.061*	
C2	0.59366 (19)	0.68218 (9)	0.5898 (2)	0.0591 (4)	
H2	0.6741	0.7188	0.6381	0.071*	
C3	0.53207 (19)	0.66771 (9)	0.4322 (2)	0.0600 (4)	
H3	0.5697	0.6950	0.3732	0.072*	
C4	0.41464 (18)	0.61275 (9)	0.36193 (17)	0.0556 (4)	
H4	0.3749	0.6030	0.2555	0.067*	
C5	0.35357 (16)	0.57122 (7)	0.44610 (15)	0.0457 (3)	
C6	0.41463 (15)	0.58712 (7)	0.60612 (15)	0.0426 (3)	
C7	0.35620 (16)	0.54362 (7)	0.69924 (15)	0.0440 (3)	
H7	0.3418	0.4926	0.6783	0.053*	
C8	0.32126 (15)	0.56882 (7)	0.81078 (15)	0.0426 (3)	
C9	0.26659 (17)	0.51217 (7)	0.88834 (15)	0.0470 (3)	
C10	0.12384 (16)	0.61012 (7)	0.94804 (14)	0.0428 (3)	
C11	0.00149 (17)	0.62388 (8)	0.99062 (16)	0.0507 (3)	
H11	−0.0461	0.5848	1.0190	0.061*	
C12	−0.04862 (17)	0.69606 (8)	0.99036 (17)	0.0506 (3)	
H12	−0.1301	0.7056	1.0200	0.061*	
C13	0.01926 (16)	0.75525 (8)	0.94698 (16)	0.0468 (3)	
C14	0.14082 (16)	0.73905 (7)	0.90318 (16)	0.0456 (3)	
H14	0.1866	0.7780	0.8724	0.055*	
C15	0.19582 (15)	0.66685 (7)	0.90391 (14)	0.0407 (3)	
C16	0.33081 (18)	0.64851 (7)	0.86226 (17)	0.0477 (3)	
H16A	0.3215	0.6813	0.7784	0.057*	
H16B	0.4379	0.6573	0.9528	0.057*	
C17	−0.0374 (2)	0.83405 (9)	0.9463 (2)	0.0661 (4)	
H17A	−0.1247	0.8461	0.8435	0.099*	
H17B	0.0540	0.8677	0.9733	0.099*	
H17C	−0.0777	0.8388	1.0216	0.099*	
C18	0.22451 (19)	0.51259 (9)	0.36486 (18)	0.0593 (4)	
H18A	0.2632	0.4656	0.4177	0.089*	
H18B	0.2034	0.5080	0.2579	0.089*	
H18C	0.1240	0.5265	0.3668	0.089*	
O1	0.17722 (13)	0.53607 (5)	0.95982 (12)	0.0540 (3)	
O2	0.29341 (16)	0.44612 (6)	0.89344 (14)	0.0657 (3)	

### Atomic displacement parameters (Å^2^)

**Table:**

	*U*^11^	*U*^22^	*U*^33^	*U*^12^	*U*^13^	*U*^23^
C1	0.0488 (7)	0.0557 (8)	0.0530 (8)	−0.0030 (6)	0.0276 (6)	−0.0087 (6)
C2	0.0545 (8)	0.0572 (9)	0.0757 (10)	−0.0044 (6)	0.0391 (8)	−0.0037 (7)
C3	0.0585 (8)	0.0652 (10)	0.0695 (10)	0.0134 (7)	0.0409 (8)	0.0153 (8)
C4	0.0566 (8)	0.0666 (9)	0.0459 (7)	0.0176 (7)	0.0260 (7)	0.0064 (7)
C5	0.0444 (6)	0.0468 (7)	0.0454 (7)	0.0118 (5)	0.0206 (6)	−0.0015 (5)
C6	0.0424 (6)	0.0421 (6)	0.0455 (7)	0.0048 (5)	0.0225 (5)	−0.0027 (5)
C7	0.0457 (6)	0.0388 (6)	0.0448 (7)	−0.0012 (5)	0.0190 (5)	−0.0038 (5)
C8	0.0449 (6)	0.0395 (6)	0.0417 (6)	−0.0049 (5)	0.0189 (5)	−0.0025 (5)
C9	0.0544 (7)	0.0410 (7)	0.0426 (7)	−0.0061 (5)	0.0204 (6)	−0.0018 (5)
C10	0.0501 (7)	0.0405 (7)	0.0382 (6)	−0.0091 (5)	0.0211 (5)	−0.0002 (5)
C11	0.0533 (7)	0.0544 (8)	0.0502 (7)	−0.0131 (6)	0.0290 (6)	0.0035 (6)
C12	0.0442 (7)	0.0598 (8)	0.0527 (8)	−0.0078 (6)	0.0268 (6)	−0.0023 (6)
C13	0.0427 (6)	0.0479 (7)	0.0484 (7)	−0.0060 (5)	0.0202 (6)	−0.0031 (6)
C14	0.0494 (7)	0.0411 (7)	0.0506 (7)	−0.0103 (5)	0.0271 (6)	−0.0034 (5)
C15	0.0459 (6)	0.0403 (6)	0.0389 (6)	−0.0108 (5)	0.0224 (5)	−0.0048 (5)
C16	0.0566 (7)	0.0412 (7)	0.0552 (8)	−0.0116 (5)	0.0344 (6)	−0.0090 (5)
C17	0.0584 (9)	0.0547 (9)	0.0922 (13)	0.0011 (7)	0.0413 (9)	−0.0017 (8)
C18	0.0586 (8)	0.0602 (9)	0.0509 (8)	0.0047 (7)	0.0190 (7)	−0.0121 (7)
O1	0.0731 (7)	0.0412 (5)	0.0592 (6)	−0.0058 (4)	0.0407 (5)	0.0045 (4)
O2	0.0869 (8)	0.0406 (6)	0.0736 (7)	−0.0014 (5)	0.0413 (7)	0.0036 (5)

### Geometric parameters (Å, º)

**Table:**

C1—C2	1.375 (2)	C10—C11	1.3819 (19)
C1—C6	1.3941 (18)	C10—O1	1.3972 (16)
C1—H1	0.9300	C11—C12	1.369 (2)
C2—C3	1.374 (2)	C11—H11	0.9300
C2—H2	0.9300	C12—C13	1.3871 (19)
C3—C4	1.375 (2)	C12—H12	0.9300
C3—H3	0.9300	C13—C14	1.3914 (18)
C4—C5	1.396 (2)	C13—C17	1.500 (2)
C4—H4	0.9300	C14—C15	1.3844 (18)
C5—C6	1.4014 (18)	C14—H14	0.9300
C5—C18	1.495 (2)	C15—C16	1.5016 (17)
C6—C7	1.4655 (18)	C16—H16A	0.9700
C7—C8	1.3360 (18)	C16—H16B	0.9700
C7—H7	0.9300	C17—H17A	0.9600
C8—C9	1.4788 (18)	C17—H17B	0.9600
C8—C16	1.4981 (17)	C17—H17C	0.9600
C9—O2	1.2025 (16)	C18—H18A	0.9600
C9—O1	1.3615 (17)	C18—H18B	0.9600
C10—C15	1.3804 (16)	C18—H18C	0.9600
			
C2—C1—C6	121.40 (14)	C10—C11—H11	120.5
C2—C1—H1	119.3	C11—C12—C13	121.58 (13)
C6—C1—H1	119.3	C11—C12—H12	119.2
C3—C2—C1	119.64 (15)	C13—C12—H12	119.2
C3—C2—H2	120.2	C12—C13—C14	117.79 (13)
C1—C2—H2	120.2	C12—C13—C17	121.07 (13)
C2—C3—C4	119.89 (14)	C14—C13—C17	121.13 (12)
C2—C3—H3	120.1	C15—C14—C13	122.15 (12)
C4—C3—H3	120.1	C15—C14—H14	118.9
C3—C4—C5	121.73 (14)	C13—C14—H14	118.9
C3—C4—H4	119.1	C10—C15—C14	117.57 (12)
C5—C4—H4	119.1	C10—C15—C16	119.47 (12)
C4—C5—C6	118.14 (13)	C14—C15—C16	122.95 (11)
C4—C5—C18	120.06 (13)	C8—C16—C15	111.67 (10)
C6—C5—C18	121.79 (13)	C8—C16—H16A	109.3
C1—C6—C5	119.18 (12)	C15—C16—H16A	109.3
C1—C6—C7	121.02 (12)	C8—C16—H16B	109.3
C5—C6—C7	119.74 (12)	C15—C16—H16B	109.3
C8—C7—C6	127.45 (12)	H16A—C16—H16B	107.9
C8—C7—H7	116.3	C13—C17—H17A	109.5
C6—C7—H7	116.3	C13—C17—H17B	109.5
C7—C8—C9	116.20 (12)	H17A—C17—H17B	109.5
C7—C8—C16	126.09 (12)	C13—C17—H17C	109.5
C9—C8—C16	117.71 (11)	H17A—C17—H17C	109.5
O2—C9—O1	116.54 (12)	H17B—C17—H17C	109.5
O2—C9—C8	125.65 (13)	C5—C18—H18A	109.5
O1—C9—C8	117.80 (11)	C5—C18—H18B	109.5
C15—C10—C11	121.98 (13)	H18A—C18—H18B	109.5
C15—C10—O1	121.54 (12)	C5—C18—H18C	109.5
C11—C10—O1	116.40 (11)	H18A—C18—H18C	109.5
C12—C11—C10	118.93 (12)	H18B—C18—H18C	109.5
C12—C11—H11	120.5	C9—O1—C10	121.72 (10)
			
C6—C1—C2—C3	−0.3 (2)	O1—C10—C11—C12	176.16 (12)
C1—C2—C3—C4	−0.8 (2)	C10—C11—C12—C13	0.7 (2)
C2—C3—C4—C5	0.8 (2)	C11—C12—C13—C14	0.1 (2)
C3—C4—C5—C6	0.3 (2)	C11—C12—C13—C17	179.65 (14)
C3—C4—C5—C18	179.28 (13)	C12—C13—C14—C15	−0.8 (2)
C2—C1—C6—C5	1.4 (2)	C17—C13—C14—C15	179.56 (13)
C2—C1—C6—C7	178.65 (13)	C11—C10—C15—C14	−0.14 (19)
C4—C5—C6—C1	−1.40 (18)	O1—C10—C15—C14	−176.75 (11)
C18—C5—C6—C1	179.66 (12)	C11—C10—C15—C16	178.69 (12)
C4—C5—C6—C7	−178.68 (12)	O1—C10—C15—C16	2.07 (19)
C18—C5—C6—C7	2.38 (18)	C13—C14—C15—C10	0.9 (2)
C1—C6—C7—C8	42.3 (2)	C13—C14—C15—C16	−177.90 (12)
C5—C6—C7—C8	−140.44 (14)	C7—C8—C16—C15	144.14 (13)
C6—C7—C8—C9	−179.09 (12)	C9—C8—C16—C15	−35.18 (17)
C6—C7—C8—C16	1.6 (2)	C10—C15—C16—C8	23.76 (17)
C7—C8—C9—O2	21.8 (2)	C14—C15—C16—C8	−157.48 (12)
C16—C8—C9—O2	−158.81 (14)	O2—C9—O1—C10	−173.36 (12)
C7—C8—C9—O1	−157.79 (12)	C8—C9—O1—C10	6.27 (18)
C16—C8—C9—O1	21.60 (17)	C15—C10—O1—C9	−18.82 (19)
C15—C10—C11—C12	−0.6 (2)	C11—C10—O1—C9	164.39 (12)

### Hydrogen-bond geometry (Å, º)

Cg is the centroid of the C1-C6 ring

**Table:**

*D*—H···*A*	*D*—H	H···*A*	*D*···*A*	*D*—H···*A*
C11—H11···O1^i^	0.93	2.53	3.437 (2)	167
C14—H14···*Cg*^ii^	0.93	2.88	3.611 (2)	137
C18—H18*A*···*Cg*^iii^	0.96	2.74	3.490 (2)	136

Symmetry codes: (i) −*x*, −*y*+1, −*z*+2; (ii) *x*, −*y*+1/2, *z*−1/2; (iii) −*x*+1, −*y*+1, −*z*+1.
